# Hyperglycemia Decreases Epithelial Cell Proliferation and Attenuates Neutrophil Activity by Reducing ICAM-1 and LFA-1 Expression Levels

**DOI:** 10.3389/fgene.2020.616988

**Published:** 2020-12-18

**Authors:** Dongxu Qiu, Lei Zhang, Junkun Zhan, Qiong Yang, Hongliang Xiong, Weitong Hu, Qiao Ji, Jiabing Huang

**Affiliations:** ^1^Xiangya Hospital, Central South University, Changsha, China; ^2^Department of Geriatrics, The Second Hospital of Xiangya, Hunan, China; ^3^Department of Cardiology, The Second Affiliated Hospital of Nanchang University, Nanchang, China; ^4^The Second Affiliated Hospital of Nanchang University, Nanchang, China

**Keywords:** hyperglycemia, ICAM-1, LFA-1, neutrophil, phagocytosis

## Abstract

Delayed repair is a serious public health concern for diabetic populations. Intercellular adhesion molecule 1 (ICAM-1) and Lymphocyte function-associated antigen 1 (LFA-1) play important roles in orchestrating the repair process. However, little is known about their effects on endothelial cell (EC) proliferation and neutrophil activity in subjects with hyperglycemia (HG). We cultured ECs and performed a scratch-closure assay to determine the relationship between ICAM-1 and EC proliferation. Specific internally labeled bacteria were used to clarify the effects of ICAM-1 and LFA-1 on neutrophil phagocytosis. Transwell assay and fluorescence-activated cell sorting analysis evaluated the roles of ICAM-1 and LFA-1 in neutrophil recruitment. ICAM-1^+^/^+^ and ICAM-1^–^/^–^ mice were used to confirm the findings *in vivo*. The results demonstrated that HG decreased the expression of ICAM-1, which lead to the low proliferation of ECs. HG also attenuated neutrophil recruitment and phagocytosis by reducing the expression of ICAM-1 and LFA-1, which were strongly associated with the delayed repair.

## Introduction

Diabetes mellitus is a chronic metabolic disorder characterized by inappropriate hyperglycemia (HG) (American Diabetes, 2013). Uncontrolled HG can lead to a host of diabetic complications, including delayed injury repair, which is a serious public health concern for subjects with diabetes. The tendon injury repair process consists of four phases: coagulation, inflammation, granular tissue formation, and remodeling (Gosain and DiPietro, 2004; Falanga, 2005). All the phases rely strongly on cellular and metabolic components of the inflamed microenvironment. However, the diabetic injury microenvironment is hostile and characterized by markedly elevated levels of inflammatory cytokines, which contribute to the dysfunction of these components. Recent studies have shown that endothelial cell (EC) proliferation at sites of injury is crucial for injury repair. Specifically, intercellular adhesion molecule 1 (ICAM-1) plays a key role in EC proliferation (Nagaoka et al., 2000; Gay et al., 2011; Sumagin et al., 2016). ICAM-1 regulates EC permeability in inflamed tissues by inducing the activation of extracellular signal-regulated kinase 1/2 (ERK1/2) (Han et al., 2016). However, due to the complexity of the immune response under HG conditions, the potential effects of ICAM-1 on ECs remain poorly understood. In the present study, we cultured ECs and performed a scratch-closure assay to evaluate the effects of ICAM-1 on EC proliferation.

Bacteria at injury sites can cause tissue infection and generate biofilms, leading to delayed injury repair (Hurlow et al., 2015; Qiu et al., 2018, 2019). HG is considered the best culture condition for bacterial growth (Frykberg, 2002). HG increases pathogen accumulation. This in turn prevents the proliferation of keratinocytes and angiogenesis at the site of injury (Gosain and DiPietro, 2004; Bandyk, 2018). Both type 1 and type 2 diabetes cause HG, indicating a high risk of insufficient injury repair within the diabetic population. Neutrophils are the main leukocytes involved in the defense against invasion by exogenous pathogens (Everett and Mathioudakis, 2018). Enhanced recruitment of neutrophils promotes injury repair in subjects with HG. Lymphocyte function-associated antigen 1 (LFA-1) is an integrin that is mainly expressed on the surface of lymphocytes (Xingyuan et al., 2006). ICAM-1 and LFA-1 expression levels are critical for neutrophil trafficking into inflamed tissues (Basit et al., 2006). However, the effects of ICAM-1 and LFA-1 on neutrophil recruitment in subjects with HG remain poorly understood. Recent studies have strongly associated neutrophil phagocytosis with ICAM-1/LFA-1 interaction (Lefort and Ley, 2012; Woodfin et al., 2016).

In the present study we explored the effects of ICAM-1 and LFA-1 on neutrophil recruitment and phagocytosis under HG conditions *in vitro* and *in vivo*. The objectives of this study were to evaluate ICAM-1 expression as well as its involvement in EC proliferation and the combined effects of LFA-1 on neutrophil recruitment and phagocytosis under HG conditions. The findings provide new insights and will inform novel therapeutic approaches for the repair of diabetic injuries.

## Materials and Methods

### Cell Culture

Injured tissue from C57BL/6 mice was harvested for EC isolation. The tissue was minced into pieces 0.3–0.4 mm in size. The pieces were enzymatically digested with trypsin and collagenase (Witkowska and Borawska, 2004). Neutrophils were freshly isolated from bone marrow. Overlying muscle and skin were removed from the tibia and femur, and the tissue was placed in Hank’s Balanced Salt Solution (HBSS) buffer on ice until needed. Bone marrow tissue was flushed with fresh HBSS for 8 min using a 10 mL sterile syringe. After rinsing, a single-cell suspension was obtained by careful pipetting. ECs and neutrophils were isolated using a magnetic separator. ECs were characterized by CD105 and CD31. Neutrophils were characterized by CD45, Ly6G, and CD11b. ECs were cultured in 500 mL complete mouse endothelial cell medium with a kit (Cell Biologics Inc., Chicago, IL, United States) supplemented with 15% fetal bovine serum (FBS; Hyclone, Logan, UT, United States), 2 mM L-glutamine, 100 mg/mL heparin, 15 mg/mL EC growth supplement, 100 mg/mL streptomycin, and 100 U/mL penicillin. Cells were grown at 37.5°C in an atmosphere of 5% CO_2_ and 95% relative humidity, and seeded in a wells of a 24-well culture plate at a density of 2 × 10^5^ cells/well.

### Scratch-Closure Assay

ECs were pre-treated with high (25 mM) or low (5 mM) glucose concentrations for 6 days. In some cases, anti-ICAM-1/LFA-1 neutralizing antibody (ab109361, ab52895; Abcam, Cambridge, MA, United States) was added to the culture medium and confluent monolayer cells were scraped off using a 200-μL pipette tip. For the *in vitro* assay, we gently removed the debris, cleaned the scratch border and replaced the volume with growth medium (Becker et al., 1991). To determine the number of ECs that had migrated into the scraped area, photographs were taken at various times and analyzed using NIS-Elements D image analysis software (Nikon, Tokyo, Japan).

### EC Proliferation Assay

EC proliferation was detected using 5-ethynyl-2-deoxyuridine (EdU) with a Click-iT Cell Proliferation imaging kit (Thermo Fisher Scientific, Waltham, MA, United States). Briefly, the indicated cells were cultured in triplicate in 24-well plates for 24 h and were then treated with 50 μM of EdU for 2 h at 37°C. Then they were fixed in 4% formaldehyde for 10 min and permeabilized with 0.5% Triton X-100 for 10 min at room temperature, the cells were treated with 1 × Apollo reaction cocktail for 30 min. For *in vitro* analysis, ECs were pre-treated with a low (5 mM) or high (25 mM) concentration of glucose and incubated with anti-ICAM-1 neutralizing antibody (15 μg/mL) or isotype IgG as a control. At least six random fields per subgroup were measured in three parallel assays. The data are expressed as the percentage of all proliferating cells in a single field. Triplicate technical replicates were assigned to each group.

### Transwell Migration Assay

Confluent ECs were continuously stimulated for 18 h with tumor necrosis factor-alpha (TNF-α) to induce EC activation prior to transmigration assays (Campos, 2012; Kolluru et al., 2012). Confluent neutrophils were inoculated into the upper chambers of the Transwell system. ECs were also added into the lower chamber of the device and incubated in fresh medium. In some cases, anti-ICAM-1 neutralizing antibody was added to the culture medium. Transwell inserts were incubated at 37.5°C in an atmosphere of 5% CO_2_ and 95% relative humidity for 20 h. Cells that migrated to the lower side of the membrane were attached, fixed with 2% paraformaldehyde (PFA) and stained with 0.5% crystal violet. Cells at the upper side of the membrane were scraped off using a cotton swab. Digital images were obtained using a light microscope system.

### Western Blotting

Skin injury tissue was isolated using the Mammalian Cell Lysis Kit (Sigma-Aldrich, St. Louis, MO, United States). Samples were adjusted to equal total protein amounts and transferred to polyvinylidene fluoride or polyvinylidene difluoride membranes. Membranes were blocked with 5% (wt/vol) blocking reagent (Roche, Basel, Switzerland) in Tris-buffered saline for 1 h. The blots were probed with rabbit monoclonal anti-ICAM-1/CD11a and β-actin antibodies (Thermo Fisher Scientific). Alkaline phosphatase conjugated to goat anti-mouse/rabbit IgG (Abcam) was added as the secondary antibody after incubation with the primary antibody.

### Animal Model

ICAM-1^+^/^+^ and ICAM-1^–^/^–^ mice were obtained from the Jackson Laboratory (Bar Harbor, ME, United States). All mice were housed under specific pathogen-free conditions in full compliance with the Animal Use and Care Committee of Central South University, Changsha, Hunan Province, China. A type 1 diabetic model was induced by continuous low-dose streptozotocin (STZ) intraperitoneal injection (50 mg/kg; Sigma-Aldrich) for 5 days. The normal control (NG) was injected with an identical dose of phosphate-buffered saline (PBS). Mice were identified as diabetic based on a blood glucose level > 250 mg/dL. Skin injury was performed after the mice had maintained a diabetic status for longer than 3 weeks. Prior to surgery, mice were anaesthetized by intraperitoneal injection with a ketamine–xylazine solution (80 mg/kg ketamine, 5 mg/kg xylazine). We used a 3.0-mm biopsy punch to perform symmetrical full-thickness excisional injury on the skin. Mice were euthanized with CO_2_ and injured tissue was collected 4 and 8 days after surgery. Seven mice per group were analyzed at each time point.

### Histology and Immunofluorescence Staining

Collected samples were fixed with formalin (10%; Sigma-Aldrich) for 20 h at 4°C, followed by slow decalcification in 10% EDTA solution for 4 weeks. Each specimen was bisected evenly, and half of the tissues were embedded in paraffin blocks for histological analysis. Slices of 5 μm thickness were prepared for hematoxylin and eosin (H&E) staining. For ICAM-1 analysis, paraffin slides were subjected to immunofluorescence staining. Slides were incubated with an ICAM-1 monoclonal antibody (Thermo Fisher Scientific) at 4°C overnight. The slide was mounted with 4,6-diamidino-2-phenylindole (DAPI) for nuclear counterstaining. Histomorphometry of the injured tissue was performed using a Nikon digital camera coupled to a microscope, followed by analysis using the associated Nikon AR software.

### Flow Cytometry Analysis

The tissue surrounding the injury edge was collected using a 4-mm punch and minced into pieces 0.1–0.2 mm in size. The pieces were transferred to conical tubes containing 5 mL digestion medium (collagenase type IV, DNase, and dispase II). The suspensions were transferred to a shaking incubator (200 rpm) at 37°C for 1 h after digestion. A 70 μm strainer was used to filter the suspended solution after shaking. The solution was centrifuged at 4°C and 400 × g for 8 min. The supernatant was removed, the pellets were resuspended in 150 μL washing buffer (3% FBS RPMI) and the cells were counted. Fluorescence-labeled murine monoclonal antibodies were obtained from BioLegend (San Diego, CA, United States) and eBioscience (San Diego, CA, United States). The isolate solution was dispensed in flow cytometry tubes (100 μL/tube). Anti-CD16/CD32 antibodies (Fc blocker; BioLegend) and 2c/100 μL cells were added for 10 min. A master mix containing CD45-Pacific Blue, CD11b allophycocyanin (APC) and Ly6G^+^ APC was created as a neutrophil panel. The mixture was centrifuged at 300 × g for 8 min at 4°C and the supernatant was gently removed. PBS was added to a volume of 200 μL and run the flow for these samples. To detect neutrophil phagocytic function, diabetic mice were intraperitoneally injected with lipopolysaccharide (LPS) to induce neutrophilia. Fluorescent zymosan-Texas-Red (ZymTR) or PBS was administered to the mice 8 and 16 h prior to tissue collection. The enzymatically digested injured tissue was analyzed by flow cytometry.

### Neutrophil Phagocytosis Assay

Bacterial phagocytosis was induced as described previously (Habas and Shang, 2018), with some modifications. Briefly, a suspension of 5 × 10^6^ neutrophils/mL was co-cultured with Staphylococcus aureus labeled with carboxy fluorescein succinimides (CFSE; Thermo Fisher Scientific). Add 50 μL of HBSS to at least one tube to create a negative (i.e., no bacteria) control for flow cytometry gating. Mix solutions very gently by inverting tubes several times. Place tubes in an incubated oven and rotate very gently (∼5–10 rpm) for 10 min. Remove tubes from incubator and immediately place on ice to arrest the phagocytosis process. Immediately add 0.55 mL of cold 4% paraformaldehyde to each tube, mix gently by inverting tubes, and incubate on ice for 30 min. Rinse cells once with cold HBSS (no Ca/Mg) by centrifugation at 400 × g for 10 min. Resuspend cells in 0.2 mL of cold HBSS (no Ca/Mg). Measure cell-associated fluorescence by flow cytometry. Neutrophil-associated bacterias were evaluated by co-localizing CFSE-labeled S. aureus. Internalized bacteria and neutrophils associated with the labeled bacteria were counted using fluorescent microscopy.

### Quantitative Real-Time Polymerase Chain Reaction (qPCR) Analysis

We performed qPCR analysis to detect the expression of ICAM-1 and LFA-1 (CD11a). Four copies per sample were analyzed and the results were averaged. The following primers were used for the PCR reactions: ICAM-1, forward primer: TTCAAGCTGAGCGACATTGG; reverse primer: CGCTC TGGGAACGAATACACA; matrix metalloproteinase-1 (MMP-1), forward primer: AGCTAGCTCAGGATGACATTGATG; reverse primer: GCCGATGGGCTGGACAG; MMP-2, forward primer: TGGCGATGGATACCCCTTT; reverse primer: TCCTCCCAAGGTCCATAGCTCAT and MMP-9, forward primer: CCTGGGCAGATTCCAAACCT; reverse primer: GCAACTCTTCCGAGTAGTTTCCAT.

### Statistical Analyses

All data are expressed as mean ± standard deviation (SD). Differences were assessed using Student’s *t*-test or paired one-way analysis of variance (ANOVA) using GraphPad Prism ver. 4.0 software (GraphPad, La Jolla, CA, United States). Statistical significance was indicated at *P* < 0.05.

## Results

### HG Reduces ICAM-1 Expression

**FIGURE 1:**
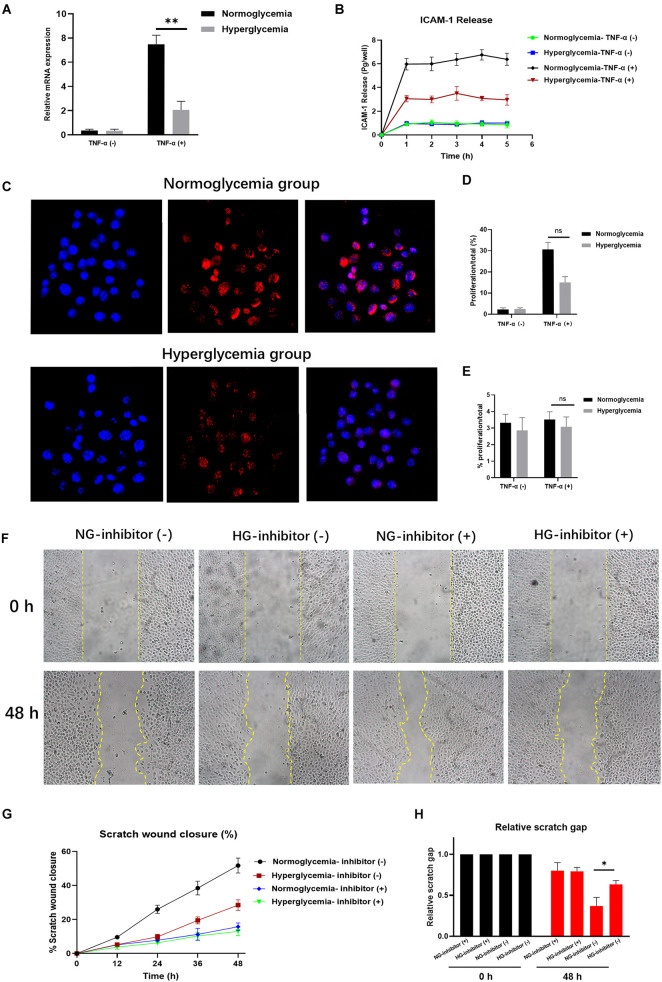
Hyperglycemia (HG) reduces ICAM-1 expression and attenuates endothelial cell (EC) proliferation. **(A)** ICAM-1 expression was lower in the HG group (*P* < 0.01). No significant differences were detected in their non-activated counterparts (NG; *P* > 0.05). **(B)** The total amount of ICAM-1 released into the basolateral chamber was decreased in the HG group. **(C)** EC proliferation was decreased in HG cultural medium. The contrast nuclear was stained with DAPI (blue) and presented in the left side. The cell proliferation was measured by 5-Ethynyl-2’-deoxyuridine (EdU) staining (red) and presented in the middle. White arrows indicate positive staining of EdU. The merged images were presented in the right. Images in upper side were from normoglycemia group. Images in lower side were from hyperglycemia group. **(D)** The expression of EdU was reduced in HG group, which indicated the low proliferation in HG culture medium. Little proliferation occurred in the absence of exogenous stimulation. **(E)** Proliferation rates declined markedly following exposure to an ICAM-1 inhibitor in the NG group. **(F,G)** In the HG group, the closure area was decreased at both 24 and 48 h post-scratching compared with the NG group. **(H)** The scratch gap distance tended to be wider in the HG group. The yellow line demarcates the closure area after scratching. Bars represent mean ± SD. *P < 0.05; **P < 0.01.

ECs increase the release of ICAM-1 during inflammation (van der Zijpp et al., 2003; Awla et al., 2011). However, little is known about the expression of ICAM-1 by ECs in subjects with HG. Since HG has been causally associated with endothelial dysfunction (Prokopowicz et al., 2012; Rada, 2019), we explored the release of ICAM-1 under hyperglycemic conditions. ECs were cultured and incubated for 16 h together with stimulation by the TNF-α pro-inflammatory cytokine. ICAM-1 expression was decreased in the HG group. However, no significant differences were detected among the non-activated counterparts (Figure 1A). To better assess ICAM-1 release by ECs, we repeated this assay using a Transwell system, which allowed us to measure levels of ICAM-1 in independent culture media. ECs were seeded as a monolayer on the Transwell interfaces. The total amount of ICAM-1 decreased in the basolateral chamber in the HG group. The rate of ICAM-1 increase was also lower in this group (Figure 1B). We inferred that MMPs are involved in ICAM-1 expression in response to ECs. To identify the effects of MMPs on the induction of ICAM-1 expression in the HG group, we focussed specifically on MMP-9, MMP-1, and MMP-2, which have been associated with ICAM-1 expression. In contrast to previous findings, no difference was detected in the levels of these MMPs between the HG and control groups (Supplementary Figures S1A–C). Based on these observations, HG appeared to reduce the release rate of ICAM-1 *in vitro*.

### HG Decreases EC Proliferation by Reducing ICAM-1 Expression

Recent studies have demonstrated the critical role played by ICAM-1 in EC proliferation (Tamanini et al., 2003; Dragoni et al., 2017). However, little is known about the effects of ICAM-1 on EC proliferation under hyperglycemic conditions. Using NG and HG culture media with or without TNF-α stimulation, we observed decreased EC proliferation in the HG culture medium as the expression of EdU was reduced in that group. Accordingly, little proliferation occurred in the absence of exogenous stimulation (Figures 1C,D). This result was verified by the introduction of anti-ICAM-1 neutralizing antibody. ICAM-1 levels decreased in both the NG and HG groups (Supplementary Figure S1D), indicating the efficiency of ICAM-1 inhibition. Notably, the proliferation rate markedly declined in the NG group following exposure to the ICAM-1 inhibitor. No significant differences were detected between the NG and HG groups (Figure 1E). Thus, EC proliferation decreased in the hyperglycemic condition through reduced expression of ICAM-1. These findings established that increased EC proliferation is a major step in injury repair (Liang et al., 2007; Bourland et al., 2019). Given our observation that HG reduced EC proliferation via ICAM-1, we hypothesized that low levels of ICAM expression would decrease injury closure in the HG group. To test this hypothesis, we introduced anti-ICAM-1 neutralizing antibody to scratch-closure EC monolayers. The effects were evaluated in terms of scratch-closure area and gap distance. The closure area was less in the HG group at both 24 and 48 h post-scratching (Figures 1F,G). The gap distance tended to be wider than that of the NG group (Figure 1H), whereas no differences in scratch-closure area or gap distance were observed following treatment with the ICAM-1 inhibitor. The collective findings indicated that injury closure was markedly delayed in the HG group due to reduced ICAM-1 expression.

### HG Attenuates Neutrophil Migration via ICAM-1 and LFA-1

**FIGURE 2:**
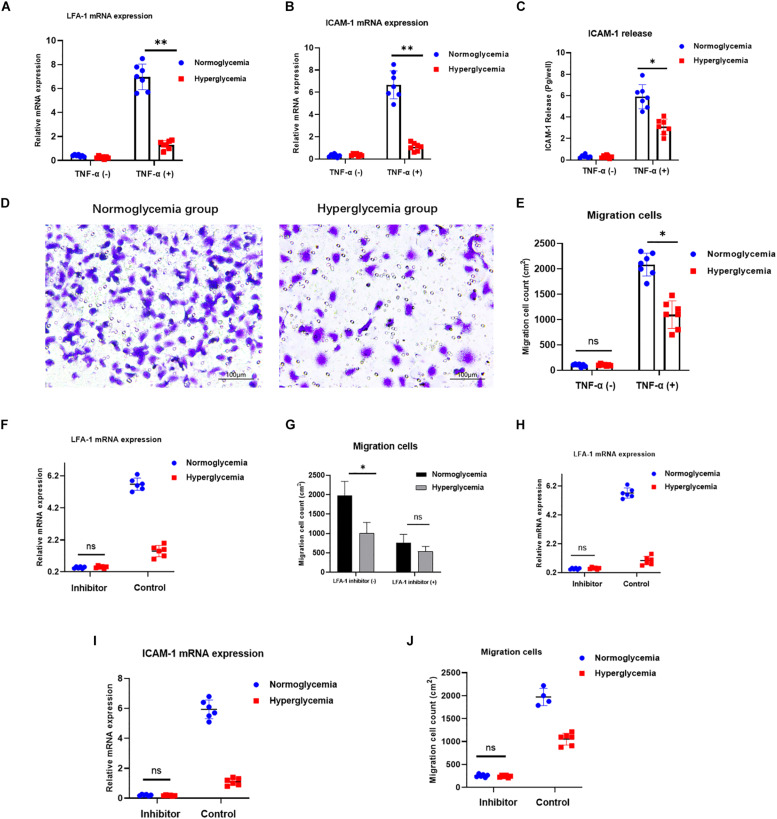
HG attenuates neutrophil migration via ICAM-1 and LFA-1. **(A–C)** Expression of ICAM-1 and LFA-1 was decreased in the HG group. **(D,E)** A Transwell migration assay revealed lower numbers of migrating neutrophils in the HG group. Images in left side were from normoglycemia group. Images in right side were from hyperglycemia group. **(F)** LFA-1 expression decreased sharply in both the NG and HG groups following exposure to the LFA-1 inhibitor. **(G)** The number of migrating neutrophils in the NG group was halved following exposure to the LFA-1 inhibitor. **(H,I)** ICAM-1 and LFA-1 expression levels decreased in both the HG and NG groups. **(J)** Little migration was observed in either group following administration of the ICAM-1 inhibitor (*P* > 0.05). Bars represent mean ± SD. *P < 0.05; **P < 0.01.

Neutrophil recruitment is strongly associated with bacterial clearance at injury sites. Therefore, efficient neutrophil migration is an essential step in injury repair. ICAM-1/LFA-1 interaction stimulates signaling pathways involved in neutrophil migration to the inflamed tissue (Lefort and Ley, 2012). To explore the effects of ICAM-1 and LFA-1 on neutrophil migration under hyperglycemic conditions, we modeled neutrophil migration under inflammation using the Transwell system. Both ICAM-1 and LFA-1 were decreased in the HG group (Figures 2A–C). Concomitantly, the Transwell migration assay revealed fewer migrating neutrophils in the HG group (*P* < 0.03) (Figures 2D,E). To independently explore the role of LFA-1 in neutrophil migration, we introduced an LFA-1 inhibitor to block the function of LFA-1. As expected, the level of LFA-1 sharply decreased in both the NG and HG groups (Figure 2F). Strikingly, the number of migrating neutrophils in the NG group was halved following exposure to the LFA-1 inhibitor (Figure 2G). To elucidate the crosslink between LFA-1 and ICAM-1 in neutrophil migration, we blocked the function of ICAM-1 using anti-ICAM-1 neutralizing antibody. As expected, the expression of LFA-1 and ICAM-1 were both reduced (Figures 2H,I) and little migration was detected in either group following the administration of the ICAM-1 inhibitor (Figure 2J). These findings indicated that HG attenuates neutrophil migration via ICAM-1 and LFA-1.

### ICAM-1 and LFA-1 Regulate Neutrophil Phagocytosis in the HG Group

**FIGURE 3:**
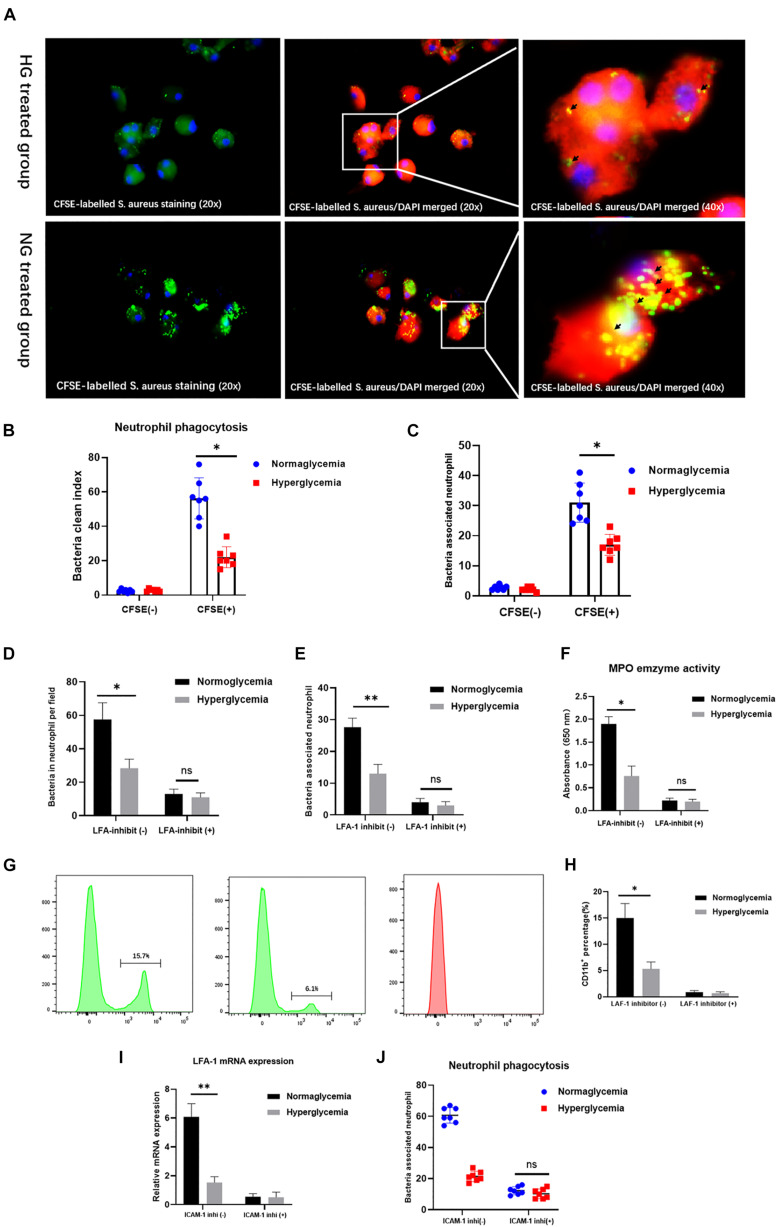
ICAM-1 and LFA-1 regulate neutrophil phagocytosis in HG culture. **(A,B)** The neutrophil clearance index was reduced in the HG group. The CFSE-labeled S. aureus staining (20x) were presented in the left side. The CFSE-labeled S. aureus/DAPI merged images (20x) were presented in the middle. Images in upper side were from hyperglycemia treated group. Images in lower side were from normoglycemia treated group. Short black arrows indicate labeled bacteria cleared by neutrophils. **(C)** The total number of positive phagocytic neutrophils associated with labeled bacteria was decreased in the HG group. **(D,E)** The number of positive phagocytic neutrophils was significantly reduced in the NG group following exposure to the LFA-1 inhibitor. **(F)** Myeloperoxidase (MPO) enzyme activity was reduced 1.2-fold in the HG group, and further decreased by exposure to the LFA-1 inhibitor. **(G,H)** CD11b expression was elevated in the NG group. However, neutrophils in the absence of LFA-1 exhibited low CD11b levels in both the NG and HG groups. **(I)** LFA-1 expression levels were decreased in both the HG and NG groups following exposure to the ICAM-1 inhibitor. **(J)** Neutrophil phagocytosis was attenuated after administration of the ICAM-1 inhibitor. Bars represent mean ± SD. *P < 0.05; **P < 0.01.

LFA-1 has been implicated in the regulation of neutrophil phagocytosis (Sigal et al., 2000). However, little is known about the role of LFA-1 in neutrophil phagocytosis under hyperglycemic conditions. Therefore, we introduced internally labeled bacteria to evaluate neutrophil phagocytosis. As exhibited in Figure 3A, the CFSE-labeled S. aureus staining were presented in the left side. The CFSE-labeled S. aureus/DAPI merged images were presented in the middle. Images in upper side were from hyperglycemia treated group. Images in lower side were from normoglycemia treated group. Neutrophil phagocytosis was evaluated by the clearance index. The results showed that the clearance index was 60% lower in the HG group (*P* < 0.05) (Figures 3A,B). Accordingly, the total number of neutrophils involved in phagocytosis of bacteria was also lower in the HG group (*P* < 0.05) (Figure 3C). These results indicated that HG attenuates neutrophil phagocytosis of bacterial pathogens. To further confirm the role of LFA-1 in neutrophil phagocytic activity, we introduced an LFA-1 inhibitor to block LFA-1 expression. The number of positive phagocytic neutrophils was significantly reduced in the NG group after exposure to the LFA-1 inhibitor (Figures 3D,E). Neutrophil activation enhances the efficiency of pathogen clearance, which is associated with the upregulation of CD11b. As shown in Figures 3G,H, CD11b expression was lower in the HG group. However, in the absence of LFA-1, neutrophils exhibited low levels of CD11b in both the NG and HG groups. Myeloperoxidase (MPO) is another neutrophil phagocytic biomarker (Muller, 2003; Ley et al., 2007). MPO generates hypochlorous acid, which aids neutrophil phagocytosis. Therefore, we extended this experiment by examining MPO activity in the NG and HG groups. An enzyme activity assay revealed that MPO activity was 1.2-fold lower in the HG group. MPO levels were further reduced in the presence of LFA-1 inhibitor (Figure 3F). To elucidate the nature of the association between LFA-1 and ICAM-1 in neutrophil phagocytosis, we blocked the function of ICAM-1 using anti-ICAM-1 neutralizing antibody. Interestingly, the expression of LFA-1was decreased in both the NG and HG groups (Figure 3I). In addition, neutrophil phagocytosis was attenuated following the administration of the ICAM-1 inhibitor (Figure 3J). These findings provided direct evidence that ICAM-1 and LFA-1 regulate neutrophil phagocytosis in hyperglycemic conditions.

### HG Decreases ICAM-1 and LFA-1 Expression *in vivo*

**FIGURE 4:**
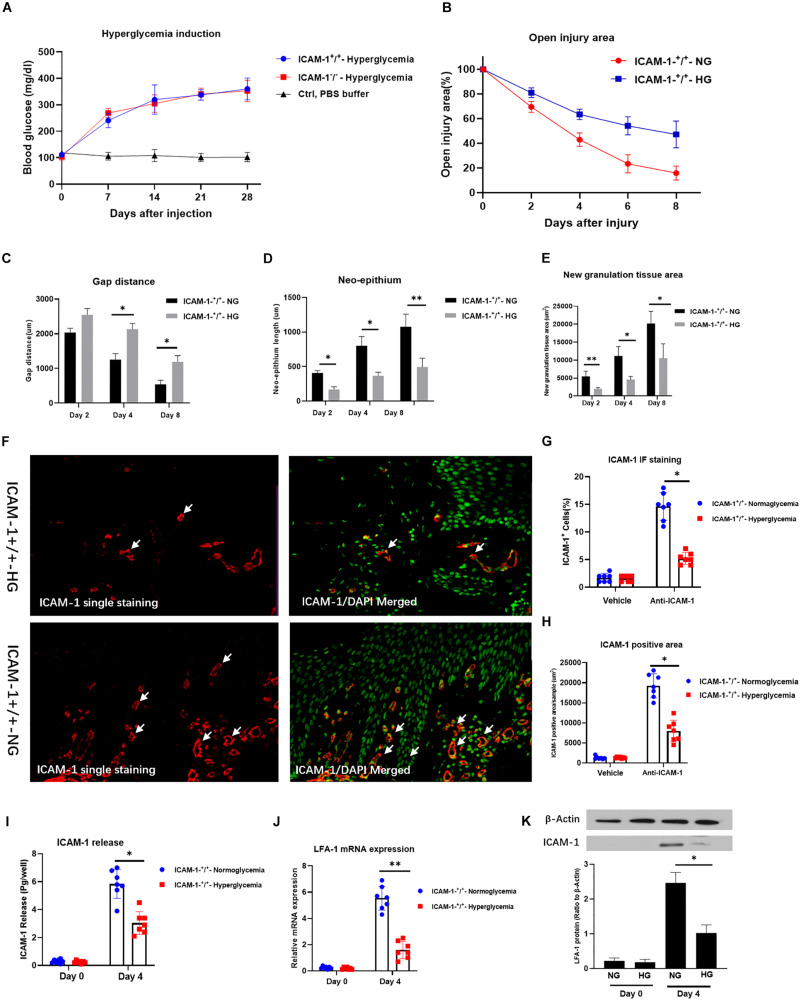
HG decreases ICAM-1 and LFA-1 expression *in vivo*. **(A)** Blood glucose levels in ICAM-1^–^/^–^-HG and ICAM-1^+^/^+^-HG mice treated by injection of streptozotocin injection. **(B)** Injury closure was significantly delayed in ICAM-1^+^/^+^-HG mice. **(C,D)** H and E staining showed that ICAM-1^+^/^+^-HG mice displayed delayed repair, incomplete re-epithelialization and larger epithelium distance. **(E)** Deposition of new granular tissue was decreased in the ICAM-1^+^/^+^-HG group. Bars represent mean ± SD. ^∗^*P* < 0.05; ^∗∗^*P* < 0.01 **(F)** Immunofluorescence (IF) analysis showed that ICAM-1 expression was decreased in ICAM-1^+^/^+^-HG injury tissue. IF staining for ICAM-1 in injury tissue were red and presented in the left side. Contrast nuclear staining were green. The merged images were presented in the right side. White arrows indicate positive staining of ICAM-1. Images in upper side were from ICAM-1^+^/^+^-HG injury group. Images in lower side were from ICAM-1^+^/^+^-NG injury group. **(G,H)** ICAM-1 expression was decreased in ICAM-1^+^/^+^-HG injury tissue **(I)** Enzyme-linked immunosorbent assay results revealed reduced release of ICAM-1 from injured tissue in ICAM-1^+^/^+^-HG mice. **(J,K)** LFA-1 expression was decreased in ICAM-1^+^/^+^-HG injured tissue. Bars represent mean ± SD. ^∗^*P* < 0.05; ^∗∗^*P* < 0.01.

To independently confirm the role of ICAM-1 and LFA-1 in the regulation of neutrophil phagocytosis *in vivo*, we induced injury in ICAM-1^+^/^+^ mice. HG was induced by continuous STZ injection for 5 days. The average blood glucose levels were 345.1 and 331.5 mg/dL in wild type (ICAM-1^+^/^+^) and ICAM-1 deletion (ICAM-1^–^/^–^) mice, respectively (Figure 4A). Skin injury repair was monitored at 2, 4, and 8 days post-surgery. Intriguingly, injury closure was significantly delayed in ICAM-1^+^/^+^HG mice (Figure 4B). Microscopy of tissues stained with H&E showed that ICAM-1^+^/^+^-HG mice displayed delayed injury repair with incomplete re-epithelialization and greater epithelium distance (Figures 4C,D). The deposition of new granular tissue was also decreased in the ICAM-1^+^/^+^HG group (Figure 4E), indicating insufficient injury repair in the hyperglycemic condition. Scratch-injury closure was markedly attenuated in the HG group due to reduced ICAM-1 expression. Immunofluorescence staining (Figures 4F–H) and ELISA analysis (Figure 4I) revealed that ICAM-1 expression was decreased in ICAM-1^+^/^+^HG injury tissue. White arrows indicated the positive staining of ICAM-1. Similarly, LFA-1 expression also decreased in ICAM-1^+^/^+^HG injury tissue (Figures 4J,K), suggesting the strong interaction between LFA-1 and ICAM-1 in injury repair *in vivo*.

### HG Impairs Neutrophil Phagocytosis and Recruitment via ICAM-1/LFA-1

**FIGURE 5:**
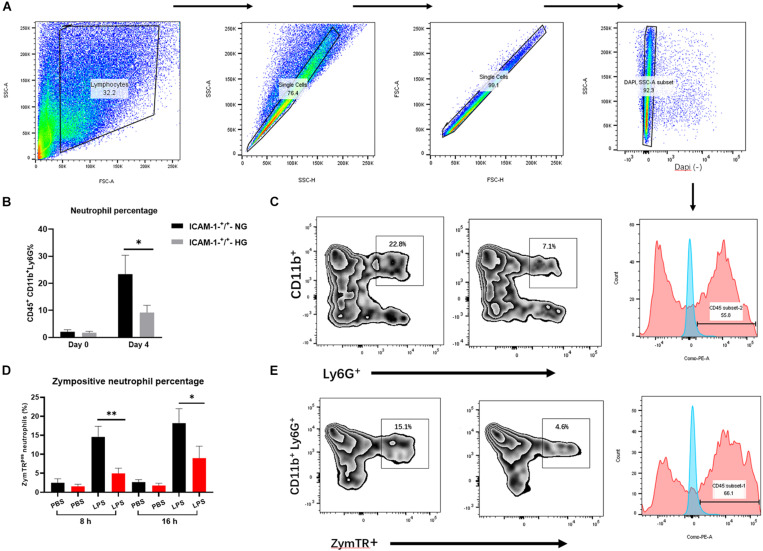
HG impairs neutrophil phagocytosis and recruitment via ICAM-1 and LFA-1 *in vivo*. **(A)** Gating strategies for neutrophils (CD45^+^CD11b^+^Ly6G^+^). **(B,C)** The proportion of neutrophils was decreased in the ICAM-1^+^/^+^-HG group. **(D,E)** The number of ZymTR positive neutrophils was reduced in the ICAM-1^+^/^+^-HG group. Bars represent mean ± SD. *P < 0.05; **P < 0.01.

**FIGURE 6:**
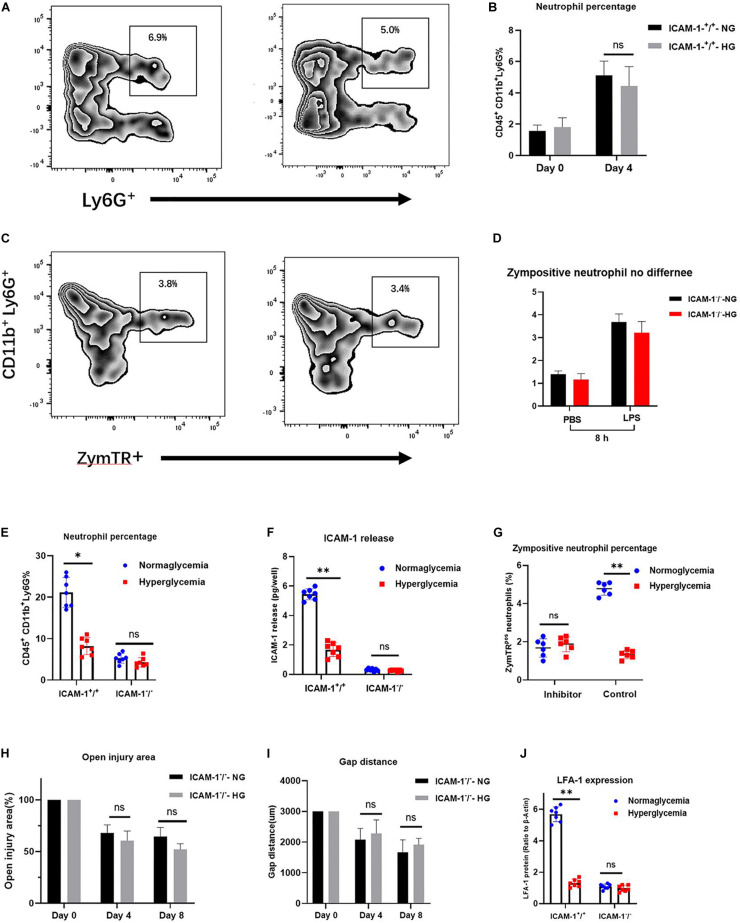
Alteration of ICAM-1 and LFA-1 expression levels attenuates neutrophil phagocytosis and decreases neutrophil recruitment in injured tissue. **(A,B)** Blocking of LFA-1 decreased neutrophil infiltration in both the ICAM-1^+^/^+^-HG and -NG groups (*P* > 0.05). **(C,D)** ZymTR positive neutrophil levels did not differ significantly between ICAM-1^+^/^+^-HG and -NG groups following administration of the LFA-1 inhibitor (*P* > 0.05). **(E)** No difference was observed between groups in ICAM-1 releasing. **(F)** Neutrophil infiltration frequency was decreased in both the ICAM-1^–^/^–^-NG and -HG groups. **(G)** ZymTR positive neutrophil levels were reduced in both the ICAM-1^–^/^–^-NG and -HG groups. **(H,I)** Open injury area and epithelium gap distance did not differ between the ICAM-1^–^/^–^-NG and -HG groups (*P* > 0.05). **(J)** LFA-1 expression was decreased in ICAM-1^–^/^–^-NG and -HG injured tissue. Bars represent mean ± SD. ^∗^*P* < 0.05; ^∗∗^*P* < 0.01.

LFA-1 has been shown to induce neutrophil migration *in vitro*. Since LFA-1 expression levels were reduced in ICAM-1^+^/^+^HG injury tissue, we hypothesized that decreased LFA-1 expression would reduce neutrophil infiltration into ICAM-1^+^/^+^HG injury sites. To evaluate neutrophil recruitment *in vivo*, Ly6G^+^ granulocytic subsets of CD11b^+^ myeloid cells were detected by fluorescence-activated cell sorting (FACS) analysis. The gating strategy used in this analysis is shown in Figure 5A. As expected, the proportion of neutrophil granulocytes (CD45^+^CD11b^+^Ly6G^+^) was decreased in the ICAM-1^+^/^+^-HG group (Figures 5B,C). As LFA-1 expression was implicated in neutrophil phagocytic activity, we also explored the effects of LFA-1 on neutrophil phagocytosis in ICAM-1^+^/^+^-HG injury tissue. The ICAM-1^+^/^+^-HG and -NG groups were treated with LPS to induce neutrophilia. Injured tissue was collected 8 and 16 h following injection of ZymTR. The number of ZymTR positive neutrophils was markedly decreased in the ICAM-1^+^/^+^HG group (Figures 5D,E). To confirm the critical role of LFA-1 in neutrophil phagocytosis and recruitment *in vivo*, we topically injected the LFA-1 inhibitor at both ICAM-1^+^/^+^-HG and ICAM-1^+^/^+^-NG injury sites. Blocking LFA-1 decreased neutrophil infiltration, with no difference detected between the ICAM-1^+^/^+^-HG and -NG groups (*P* > 0.05) (Figures 6A,B). No significant difference in ZymTR positive neutrophils was detected between the groups (*P* > 0.05) (Figures 6C,D). As described above, ICAM-1 induced LFA-1 expression and was implicated in neutrophil migration and phagocytosis *in vitro*. However, the exact interactions between ICAM-1 and LFA-1 *in vivo* required further elucidation. To clarify the ICAM-1/LFA-1 association *in vivo*, we used ICAM-1^–^/^–^ mice as an injury model. Unlike the ICAM-1^+^/^+^ mice, both the ICAM-1^–^/^–^-NG and -HG groups of mice displayed a decreased frequency of neutrophil infiltration (Figure 6E), the release of ICAM-1 was also detected by ELISA analysis, and no difference were detected between ICAM-1^–^/^–^-NG and -HG group (Figure 6F). Parallel results were observed in ZymTR positive neutrophils (Figure 6G). Thus, although injury repair was dramatically delayed in ICAM-1^–^/^–^-NG mice (Figures 6H,I), no difference was observed between groups. Notably, decreased LFA-1 expression was observed in both the ICAM-1^–^/^–^-NG and -HG groups (Figure 6J). The collective results provided direct evidence that HG affects the expression of ICAM-1 and LFA-1, which results in insufficient injury repair. Furthermore, changes in ICAM-1 and LFA-1 expression levels impair neutrophil phagocytosis and decrease neutrophil recruitment in the injured tissue.

## Discussion

ICAM-1 is a key member of the immunoglobulin superfamily and is centrally involved in EC proliferation and neutrophil trafficking (Gay et al., 2011; Sumagin et al., 2016; Qiu et al., 2020). ICAM-1 is expressed at low levels on the surface of ECs, but is upregulated in response to a variety of inflammatory cytokines. ICAM-1 has been recently implicated in the regulation of injury repair by promoting EC proliferation. However, due to the complex immune response in HG, the potential effects of ICAM-1 on EC proliferation remain unclear. While investigating the natural status of ICAM-1 release, we unexpectedly found that HG decreased ICAM-1 expression, resulting in the dramatic attenuation of EC proliferation. These findings were confirmed by introducing the ICAM-1 inhibitor to rule out other factors that might contribute to EC proliferation. Similar to the results of the ICAM-1 release assay, we found that the EC proliferation rate of the NG group was markedly attenuated following exposure to the ICAM-1 inhibitor, and injury closure was decreased by HG at 24 and 48 h following creation of the scratch assay. However, no effect was observed following exposure to the ICAM-1 inhibitor. Thus, we conclude that HG can reduce the expression of ICAM-1 and prolong injury closure *in vitro*. These findings extend our knowledge of ICAM-1 function in the HG injury repair process.

We also evaluated the potential mechanism of ICAM-1 expression regulation in HG. Previous studies reported the involvement of MMPs, including MMP-9, MMP-1, and MMP-2, in the release of ICAM-1. However, a further experiment revealed no significant differences in these MMPs between the NG and HG groups, suggesting that MMPs are unlikely to play a role in ICAM-1 expression in HG. Other important kinases, such as mitogen-activated protein kinase (MAPK), c-Jun N-terminal kinase and ERK1/2, are reportedly involved in ICAM-1 expression (Christensen and Bruggemann, 2014; Hurabielle et al., 2020). Further studies focussing on these kinases are required to elucidate the potential mechanisms of ICAM-1 expression.

LFA-1 is a heterodimeric integrin consisting of αL and β2 subunits expressed on the surface of neutrophils (Lefort and Ley, 2012). Recent studies have shown that the interaction of LFA-1 with its ligand ICAM-1 mediates several important steps in the cell immune response. For example, LFA-1 integrin is critical for the firm adhesion of neutrophils to ICAM-1 (Meisel et al., 2018) and the expression of ICAM-1 and LFA-1 triggers the activation of myosin light chains, MAPK and Rho GTPase, which enhances neutrophil transmigration into inflamed tissues (Wolcott et al., 2016; Bourland et al., 2019). Neutrophil recruitment has been associated with bacterial clearance at injury sites. Specifically, ICAM-1 and LFA-1 are essential for neutrophil trafficking to inflamed tissue. However, the impact of ICAM-1 and LFA-1 on neutrophil migration in HG remains poorly understood. In this context, we explored the causative involvement of ICAM-1 and LFA-1 by modeling neutrophil migration in an inflammatory stimulation model. As expected, both LFA-1 and ICAM-1 were attenuated in the HG medium. Intriguingly, the Transwell migration assay also revealed fewer migrating neutrophils in the HG group, which was consistent with the low expression of ICAM-1 and LFA-1. The results indicate that HG attenuates neutrophil migration by regulating the expression of ICAM-1 and LFA-1. We further identified the association between LFA-1 and ICAM-1 in neutrophil migration by blocking the function of ICAM-1. Little migration was observed in the NG or HG group following exposure to an ICAM-1 inhibitor. Together, these results provide solid evidence that, under hyperglycemic conditions, ICAM-1 is involved in neutrophil migration by inducing LFA-1 expression.

We further analyzed the role of LFA-1 in neutrophil phagocytosis by introducing internally labeled bacteria. Interestingly, in HG medium, the ability of neutrophils to clear the bacteria was dramatically attenuated and the total number of neutrophils associated with labeled bacteria was reduced. These findings indicated that HG impairs neutrophil phagocytosis. To confirm these findings, we introduced an LFA-1 inhibitor to block LFA-1 expression. Surprisingly, the number of positive phagocytic neutrophils was sharply reduced in the NG group following exposure to the LFA-1 inhibitor, while no difference was detected in the HG group. These results support the hypothesis that HG decreases neutrophil phagocytosis by reducing LFA-1 expression. The findings suggest that it may be possible to promote neutrophil phagocytic activity to enhance LFA-1 expression in subjects with HG. To elucidate the interplay between LFA-1 and ICAM-1 in neutrophil phagocytosis, we blocked the function of ICAM-1 using anti-ICAM-1 neutralizing antibody. LFA-1 expression was dramatically decreased in both groups. Neutrophil phagocytosis was also attenuated following the administration of the ICAM-1 inhibitor. Together, these findings demonstrate the interconnection between ICAM-1 and LFA-1, neutrophil phagocytosis and HG in an *in vitro* model. Both type 1 and type 2 diabetes cause HG, which contributes to the accumulation of pathogens at injury sites, leading to insufficient injury repair (Standiford et al., 1990; Cunningham and Kirby, 1995; Liang et al., 2007; Herrera et al., 2015). Enhancing neutrophil phagocytosis by the regulation of ICAM-1/LFA-1 expression may provide novel therapeutic approaches for diabetic injury repair.

To independently confirm these observations *in vivo*, we induced skin injury using ICAM-1^+^/^+^ and ICAM-1^–^/^–^ mouse models. Consistent with our *in vitro* results, ICAM-1^+^/^+^-HG mice exhibited delayed injury repair with incomplete re-epithelialization and larger epithelium distance as well as decreased neutrophil recruitment and phagocytic activity. Importantly, the frequency of neutrophil infiltration declined dramatically in ICAM-1^+^/^+^-HG injured tissue. Similar results were also obtained in ZymTR positive neutrophils, which showed decreased levels of LFA-1. Together, these findings confirmed our *in vitro* results and indicate that HG attenuates skin injury repair and decreases neutrophil phagocytosis and recruitment by regulating ICAM-1 and LFA-1 expression.

Notably, plenty of researches showed that HG increased the expression of ICAM-1 in umbilical vein as well as in microvascular endothelial cell. In disagree with the previously findings, our study obtained the opposite results indicating that HG decreased the ICAM-1 expression in HG condition. The reasons might be as following: after the skin injury, invading pathogens and necrotic debris triggers an acute inflammatory response, which contributed to the pathogen defensing and the debris removing. Unlike the situation in microvascular endothelial or umbilical vein endothelial cells, the endothelial cells in injury tissue was exposure directly to the outside environment, and the pathogen around the injury site was easily invading into the internal injury tissue (Grice et al., 2010). Thus, the acute inflammation in injury area was critical to defense against the bacterial infections. Therefore, a source of proinflammatory cytokines, including the ICAM-1, was highly expressed in injury site to response to exogenous pathogens. ICAM-1 is constitutively presented on endothelial cells and reported to be a pro-inflammatory cytokine involved in the acute inflammatory process. ICAM-1 is also critical for the firm arrest and transmigration of neutrophil out of blood vessels into the injury tissue. Neutrophils are efficiently entering tissue and enable to engulf invading pathogens. Additionally, neutrophil release antimicrobial peptides, ROS, and cytotoxic enzymes to defense against extracellular pathogens (Wolcott et al., 2008; Su and Richmond, 2015). Therefore, the expression of ICAM-1 is closely related to the recruitment of neutrophil and worked as the protective factors within the injury repair. Taken together, as the inflammatory microenvironment was distinctive and complex in injury tissue, the expression of ICAM-1 might not be the same as the previous study and could be changed according to the specific reality. However, although we obtained the valid results based on rigorous experimental design, more studies are still required to further confirm it.

## Conclusion

The scratch-closure assays of NG and HG cultured tissues demonstrated that HG decreases ICAM-1 expression, which results in low EC proliferation. A Transwell assay and FACS analysis further indicated that HG attenuates neutrophil recruitment and phagocytosis by reducing ICAM-1 and LFA-1 expression. These observations were confirmed *in vivo* in ICAM-1^+^/^+^ and ICAM-1^–^/^–^ mouse injury models. Together, these results highlight the important roles of ICAM-1 and LFA-1 in EC proliferation and neutrophil activity in HG culture. Targeting ICAM-1 and/or LFA-1 may provide an alternative approach for improving injury repair in diabetic populations.

## Data Availability Statement

The raw data supporting the conclusions of this article will be made available by the authors, without undue reservation, to any qualified researcher.

## Ethics Statement

The animal study was reviewed and approved by the Central South University of Animal Care and Use Committee.

## Author Contributions

DXQ wrote the manuscript. JBH designed and supervised the study. LZ, JKZ, QY, and HLX performed the statistical analyses. DXQ, LZ, JKZ, QY, HLX, WTH, QJ, and JBH critically revised the manuscript for intellectual content. All authors contributed to the article and approved the submitted version.

## Conflict of Interest

The authors declare that the research was conducted in the absence of any commercial or financial relationships that could be construed as a potential conflict of interest.

## Supplementary Material

Supplementary Figure 1 **(A–C)** MMP-9, MMP-1 and MMP-2 levels did not differ significantly between the NG and HG groups (*P* > 0.05). **(D)** ICAM-1 expression was decreased in both the NG and HG groups (*P* > 0.05). Bars represent mean ± SD.
