# Corrigendum: Hyperglycemia decreases epithelial cell proliferation and attenuates neutrophil activity by reducing ICAM-1 and LFA-1 expression levels

**DOI:** 10.3389/fgene.2024.1431933

**Published:** 2024-07-16

**Authors:** Dongxu Qiu, Lei Zhang, Junkun Zhan, Qiong Yang, Hongliang Xiong, Weitong Hu, Qiao Ji, Jiabing Huang

**Affiliations:** ^1^ Xiangya Hospital, Central South University, Changsha, China; ^2^ Department of Geriatrics, The Second Hospital of Xiangya, Hunan, China; ^3^ Department of Cardiology, The Second Affiliated Hospital of Nanchang University, Nanchang, China; ^4^ The Second Affiliated Hospital of Nanchang University, Nanchang, China

**Keywords:** hyperglycemia, ICAM-1, LFA-1, neutrophil, phagocytosis

In the published article, there was an error in Figure 1F as published. Figure 1F of the NG-inhibitor (+) in 48 h subgroup was incorrect. This figure belongs to the HG-inhibitor (+) group. However, it was presented in NG-inhibitor (+) group by mistake.

**FIGURE 1:**
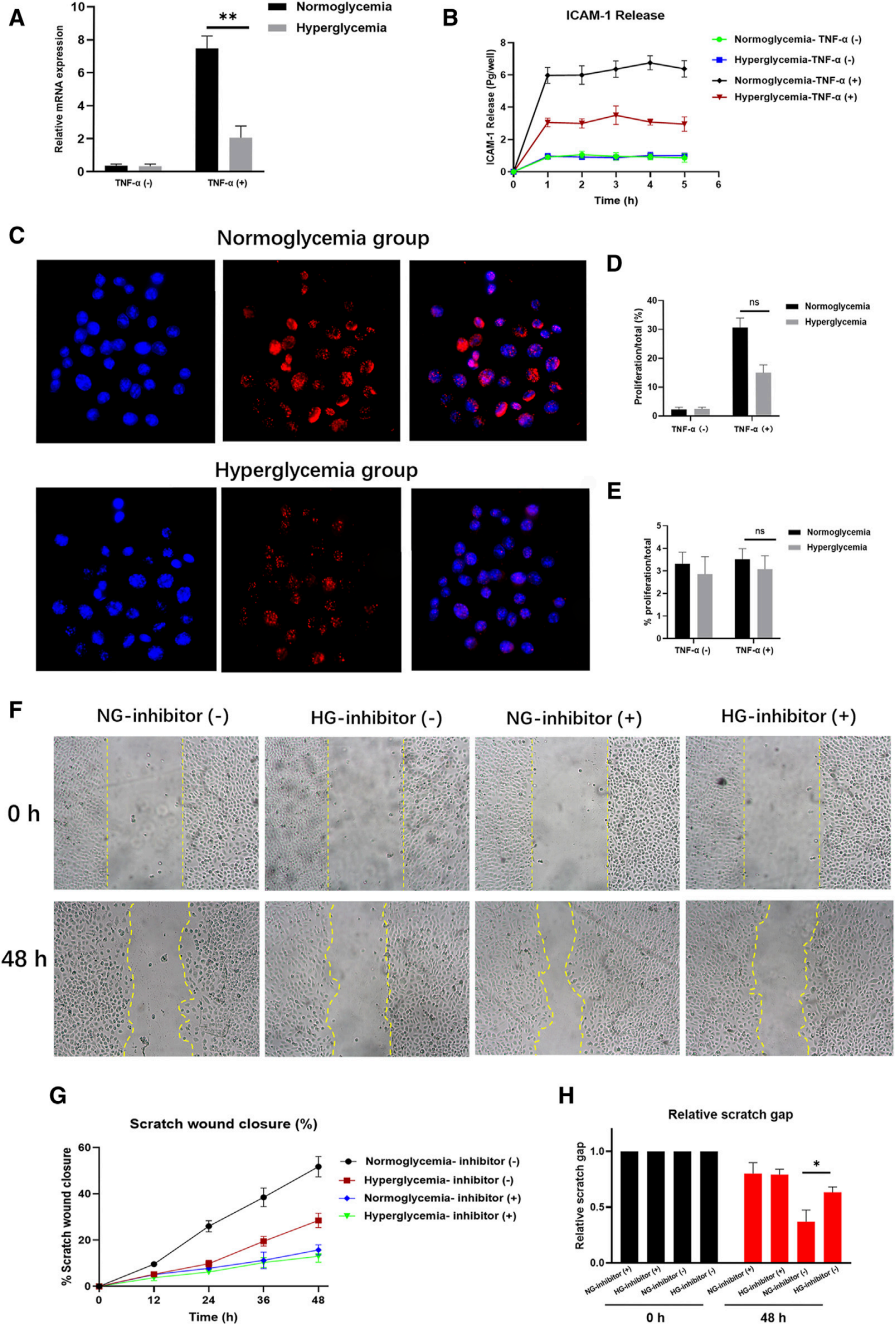
Hyperglycaemia (HG) reduces ICAM-1 expression and attenuates endothelial cell (EC) proliferation. **(A)** ICAM-1 expression was lower in the HG group (*p* < 0.01). No significant differences were detected in their non-activated counterparts (NG; *p* > 0.05). **(B)** The total amount of ICAM-1 released into the basolateral chamber was decreased in the HG group. **(C, D)** EC proliferation was decreased in HG cultural medium. **(E)** Proliferation rates declined markedly following exposure to an ICAM-1 inhibitor in the NG group. **(F, G)** In the HG group, the closure area was decreased at both 24 and 48 h post-scratching compared with the NG group. **(H)** The scratch gap distance tended to be wider in the HG group. The yellow line demarcates the closure area after scratching. Bars represent mean ± SD. *p < 0.05; **p < 0.01.

The corrected Figure 1 and its caption appear below.

The authors apologize for this error and state that this does not change the scientific conclusions of the article in any way. The original article has been updated.

