# Supplementary figures and images for: Hyperglycemia Decreases Epithelial Cell Proliferation and Attenuates Neutrophil Activity by Reducing ICAM-1 and LFA-1 Expression Levels
Source: Front Genet. 2020 Dec 18;11:616988. doi: 10.3389/fgene.2020.616988 (PMC7785031; doi:10.3389/fgene.2020.616988)

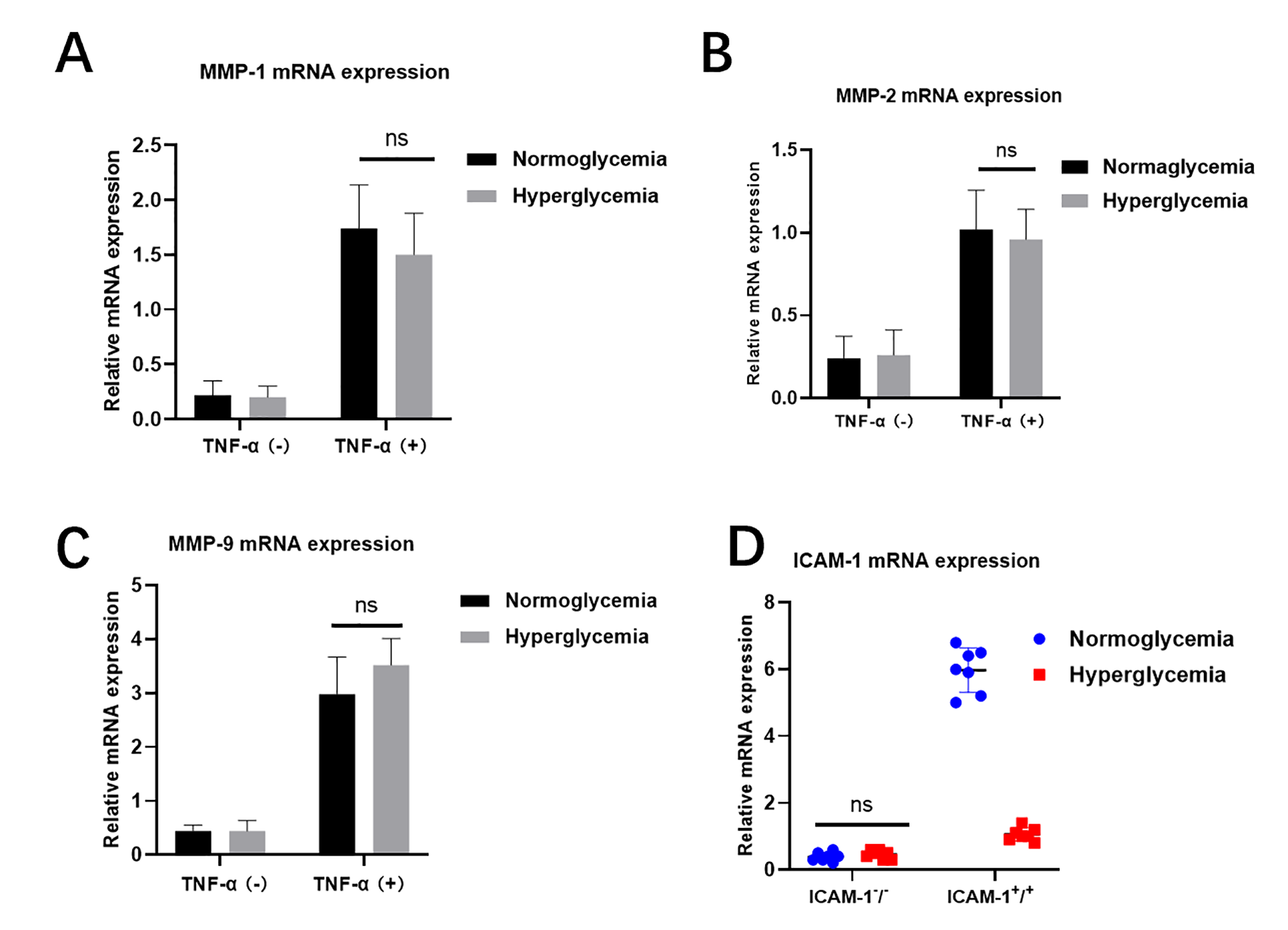

Supplement: Supplementary Figure 1 — (A–C) MMP-9, MMP-1 and MMP-2 levels did not differ significantly between the NG and HG groups (P > 0.05). (D) ICAM-1 expression was decreased in both the NG and HG groups (P > 0.05). Bars represent mean ± SD. [file Image_1.TIF]
